# Hsa-miR-19a is associated with lymph metastasis and mediates the TNF-α induced epithelial-to-mesenchymal transition in colorectal cancer

**DOI:** 10.1038/srep13350

**Published:** 2015-08-25

**Authors:** Lanlan Huang, Xiaoyan Wang, Chuangyu Wen, Xiangling Yang, Minmin Song, Junxiong Chen, Chenliang Wang, Bo Zhang, Lei Wang, Aikichi Iwamoto, Jianping Wang, Huanliang Liu

**Affiliations:** 1 Guangdong Institute of Gastroenterology and the Sixth Affiliated Hospital; 2 Guangdong Key Laboratory of Colorectal and Pelvic Floor Diseases; 3 Institute of Human Virology and Key Laboratory of Tropical Disease Control (Ministry of Education), Sun Yat-sen University Guangzhou, China; 4 Advanced Clinical Research Center, Institute of Medical Science, University of Tokyo, Tokyo, Japan

## Abstract

Lymph node metastasis is an important factor determining the outcome of colorectal cancer. Although epithelial-to-mesenchymal transition (EMT), TNF-α and microRNA (miRNA) have been found to play important roles in lymph node metastasis, the underlying molecular mechanism remains unclear. Here we reported that high expression of microRNA-19a (miR-19a) was associated with lymph node metastasis and played an important role in TNF-α-induced EMT in colorectal cancer (CRC) cells. We analyzed miR-19a expression in surgical tissue specimens from 11 CRC patients and 275 formalin-fixed, paraffin-embedded CRC patients. We found that miR-19a was up-regulated in CRC tissues and high expression of miR-19a was significantly associated with lymph node metastasis. We further analyzed miR-19a lymph node metastasis signature in an external validation cohort of 311 CRC cases of the TCGA. MiR-19a was found to be significantly associated with lymph node metastasis in rectal cancer. *In vitro*, we showed that overexpression of miR-19a in human CRC cell lines promoted cell invasion and EMT. Furthermore, miR-19a was up-regulated by TNF-α and miR-19a was required for TNF-α-induced EMT and metastasis in CRC cells. Collectively, miR-19a played an important role in mediating EMT and metastatic behavior in CRC. It may serve as a potential marker of lymph node metastasis.

Once thought of as junk DNA, microRNAs (miRNAs) are a class of non-coding RNAs that play key roles in the regulation of gene expression. MiRNAs are single-stranded RNA of 18–24 nucleotides and are generated by an RNase III-type enzyme from genomic DNAs^1^. MiRNA functions as a guide molecule in post-transcriptional gene silencing by partially complementing with the 3′-untranslated region (UTR) of target mRNAs, leading to translational repression^2^. Colorectal cancer (CRC) is one of the most prevalent malignancy tumors with high morbidity and mortality^3^. Lymph node metastasis is an important factor determining the outcome of CRC^4^,^5^. Therefore, a better understanding of the regulation mechanisms of lymph node metastasis is crucial for the development of novel diagnosis and therapeutic strategies for CRC. Accumulating evidences indicated that miRNAs serve as biomarkers for the early detection, prognosis, and determination of predictive responses to chemotherapy in patients with CRC^6^,^7^,^8^. Recent data showed that several miRNAs such as miR-21, miR-19a-3p, miR-499-5p, miR-493, miR-92a-3p and miR-422a have a potential role as biomarkers of CRC metastasis^8^,^9^,^10^,^11^.

Here we further classified the roles of miR-19a in CRC progression, invasion and metastasis. MiR-19a belongs to the miR-17-92 cluster, and genes in this cluster have been validated as widely over-expressed in diverse tumor subtypes^12^. MiR-19a is up-regulated in various cancers such as lung cancer, cervical carcinoma and gastric cancer and act as oncomiR^13^,^14^,^15^. Recently, studies reported that miR-19a promoted the proliferation and metastasis in colon cancer and cervical carcinoma^14^,^16^. These data suggested a potential function of miR-19a in carcinogenesis^12^,^13^,^14^,^15^,^16^,^17^. However, the role of miR-19a in lymph node metastasis has never been evaluated.

One of the pivotal molecular steps in the process of metastasis includes epithelial-to-mesenchymal transition (EMT). During EMT, epithelial tumor cells lose their epithelial morphology and gain of mesenchymal markers, which promote increased motility and invasiveness^18^. Several inflammatory mediators such as TGF-β, hypoxia and IL-6 can trigger EMT^14^,^19^. Previous studies have showed that high TNF-α expression was significantly associated with positive lymph node stage and recurrence of the tumor^20^. TNF-α can induce EMT and thereby promotes invasion and metastasis in CRC^21^. However, the underlying molecular mechanism is not fully understood.

Here we reported that miR-19a was up-regulated in CRC tissue and high expression of miR-19a was significantly associated with lymph node metastasis. Overexpression of miR-19a significantly enhanced cell invasion and induced EMT. In addition, we also found that miR-19a was up-regulated by TNF-α and miR-19a was required for TNF-α-induced EMT and invasion in CRC cells. Taken together, miR-19a may act as a molecular marker for lymph node metastasis in CRC patients and mediate TNF-α induced EMT of CRC cells.

## Results

### Patients

The general information of 11 cases of CRC was summarized in Table S1. Demographic and clinical information was summarized in Table S2 for 275 CRC patients.

### High levels of miR-19a expression were associated with regional lymph node metastasis in CRC

**Figure 1:**
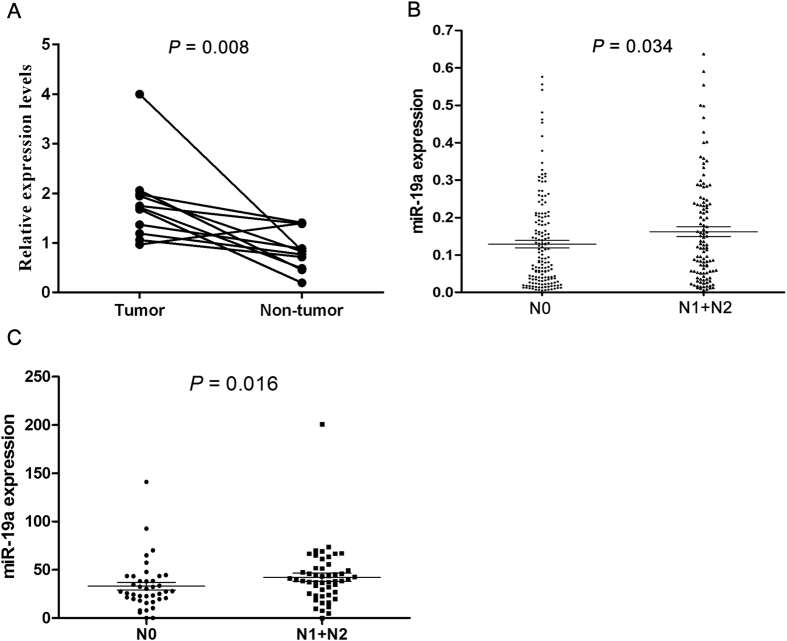
MiR-19a expression levels in CRC specimens. (**A**) TaqMan RT-PCR analysis of miR-19a expression levels in 11 paired CRC tissues and adjacent normal tissues. The CRC tissues expressed significantly higher levels of miR-19a than the adjacent normal tissues (*P* = 0.008), the median (lower quartile, upper quartile) of tumor and adjacent normal tissues group were 0.91 (0.33, 1.27) and 0.38 (0.34, 0.46) respectively. (**B**) *In situ* hybridization from paraffin blocks of CRC specimens followed by analysis of miR-19a levels. MiR-19a expression levels in CRC with lymph node metastases (n = 112) were significantly higher than in CRC without lymph node metastasis (n = 156, *P* = 0.034), the mean ± standard deviation of N0 and N1 + N2 group were 0.13 ± 0.12 and 0.16 ± 0.14 respectively. (**C**) Statistical analysis of miR-19a expression data in rectal cancer from the TCGA dataset. MiR-19a was up-regulated in rectal cancer patients with lymph node metastasis (n = 50) compared to those without metastasis (n = 40, *P* = 0.004), the median (lower quartile, upper quartile) of N0 and N1 + N2 group were 27.40 (19.61, 42.09) and 39.16 (25.01, 51.57) respectively.

Taqman RT-PCR was used to assess the expression of miR-19a in 11 pairs of CRC and adjacent non-tumorous tissues. The result showed that miR-19a was significantly up-regulated in tumor tissue compared with the corresponding normal tissues (*P* = 0.008, Fig. 1A). To further investigate the clinical pathology and prognostic significance of miR-19a expression in CRC patients, levels of miR-19a were quantified in a cohort of 275 formalin-fixed, paraffin-embedded CRC samples using *in situ* hybridization. The positive staining of tissue was expressed as blue–violet (Supplementary Fig. S1). As shown in Table 1, the expression of miR-19a was correlated with lymph node metastasis (*P* = 0.018) and TNM stage (*P* = 0.008). There was no statistically significant difference between age, gender, tumor size, tumor location, distant metastasis or relapse (*P* > 0.05, Table 1). Analysis using the X-tile software revealed that 0.22 was the optimal cut-point that separated patients into a group with miR-19a lower expression and group with miR-19a higher expression. Kaplan-Meier curve and log-rank test showed that miR-19a expression wasn’t associated with survival (*P* = 0.171, Table 2, Supplementary Fig. S2). In multivariate analysis by Cox proportional hazards model, tumor location, relapse and TNM stage were found to be the independent prognostic factors for CRC (*P* < 0.05, Table 2).

We then analyzed miR-19a lymph node metastasis signature in an external validation set. The publicly available TCGA miRNAseq data set with clinical information was downloaded on 06/19/2015. Clinical information for 218 colon cancer patients and 93 rectal cancer patients was summarized in Table S3 and S4, respectively. In colon cancer, miR-19a expression was not significantly associated with lymph node metastasis (*P* = 0.302, Table S5). However, miR-19a expression was correlated with lymph node metastasis in rectal cancer (*P* = 0.004, Table S6). MiR-19a was up-regulated in rectal cancer patients with lymph node metastasis compared to those without metastasis (Fig. 1C).

### Overexpression of miR-19a promoted CRC cells progression *in vitro*

**Figure 2:**
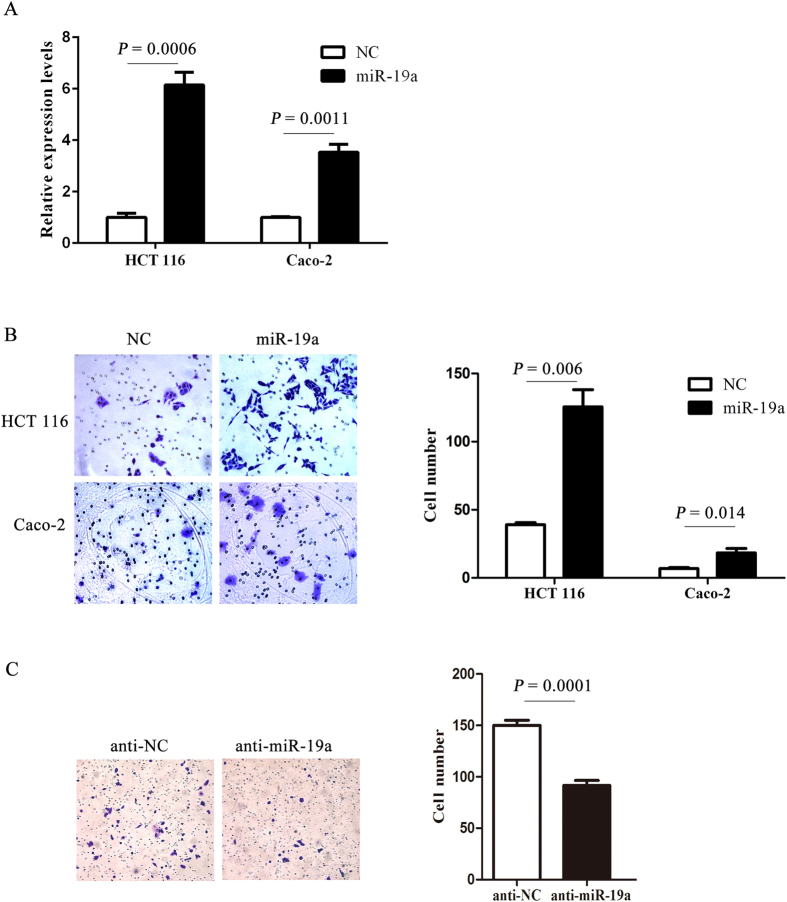
Overexpression of miR-19a enhanced invasion in CRC cells. (**A**) Real-Time PCR analysis confirmed the expression of miR-19a in HCT 116 and Caco-2 cells infected with the miR-19a lentiviral vector or the scramble control lentiviral vector. (**B**) The HCT 116 and Caco-2 cells infected with the miR-19a lentiviral vector or the scramble control lentiviral vector were subjected to invasion assay. Overexpression of miR-19a promoted invasion (*P* < 0.05). (**C**) The HCT 116 cells were transiently transfected with anti-miR-19a or negative control oligos. Transfection with anti-miR-19a inhibited CRC cells invasion (*P* = 0.0001).

**Figure 3:**
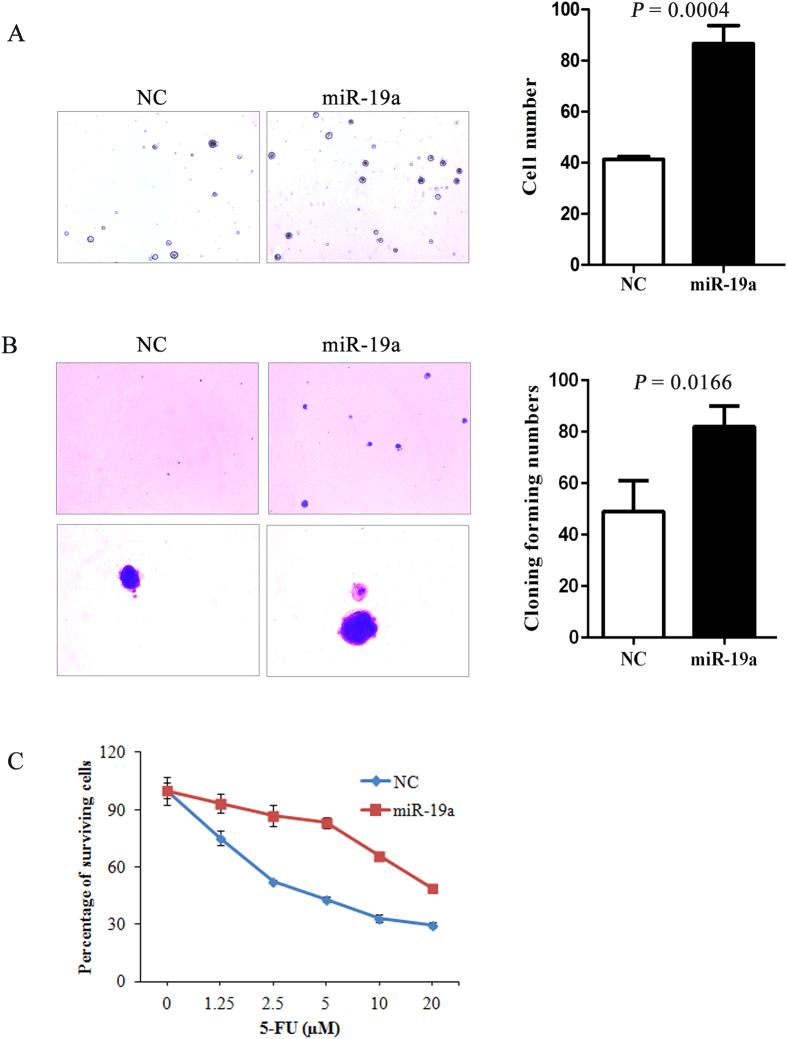
Overexpression of miR-19a promoted CRC cells progression *in vitro*. (**A**) Caco-2 expressing miR-19a formed more spheres than those transfected with the scramble control (*P* = 0.0004). (**B**) MiR-19a-transfected Caco-2 cells form more colonies with larger sizes (*P* = 0.0166). (**C**) The miR-19a- and scramble-transfected HCT 116 cells were cultured and treated with 5-FU, followed by analysis of cellular viability. Cells transfected with miR-19a displayed resistance to 5-FU.

Higher miR-19a expression was associated with lymph node metastasis, suggested that miR-19a may affect invasion of human CRC. To determine the role of miR-19a in CRC cells, HCT 116 and Caco-2 cells were stably infected with the miR-19a lentiviral vector or scramble control lentiviral vector. Increased expression of miR-19a in cells following infection was confirmed by Real-Time PCR (Fig. 2A). Invasion assays were performed to determine the effects of miR-19a on invasive capacity of CRC cells. As shown in Fig. 2B, ectopic miR-19a expression significantly enhanced the invasive ability of HCT 116 and Caco-2 cells (*P* < 0.05). When compared with the scramble control HCT 116 cells, anti-miR-19a HCT 116 transfected cells had a decrease in the number of invaded cells (*P* = 0.0001, Fig. 2C). To investigate the potential role of miR-19a in self-renewal of CRC cells, stable clones from Caco-2 cells infected with miR-19a or scramble control lentiviral vector were plated for tumorsphere culture in ultra-low adhesion plates 15 days later, tumorspheres were observed and quantified under a microscope. As shown in Fig. 3A, the miR-19a transfected cells had significant more tumorspheres as compared with that of the scramble control (*P* = 0.0004). The cells were also cultured in soft agar for colony formation assays. Caco-2 cells expressing miR-19a formed significantly more colonies than scramble control (*P* = 0.0166, Fig. 3B). MiRNA has been identified as an important regulator of chemotherapeutic treatment. Therefore, we investigated the effects of chemotherapeutic agents on CRC cells. Overexpression of miR-19a facilitated cell survival after treating with 5-Fluorouracil (5-Fu) as expected (Fig. 3C).

### Overexpression of miR-19a induced EMT in CRC cell lines

**Figure 4:**
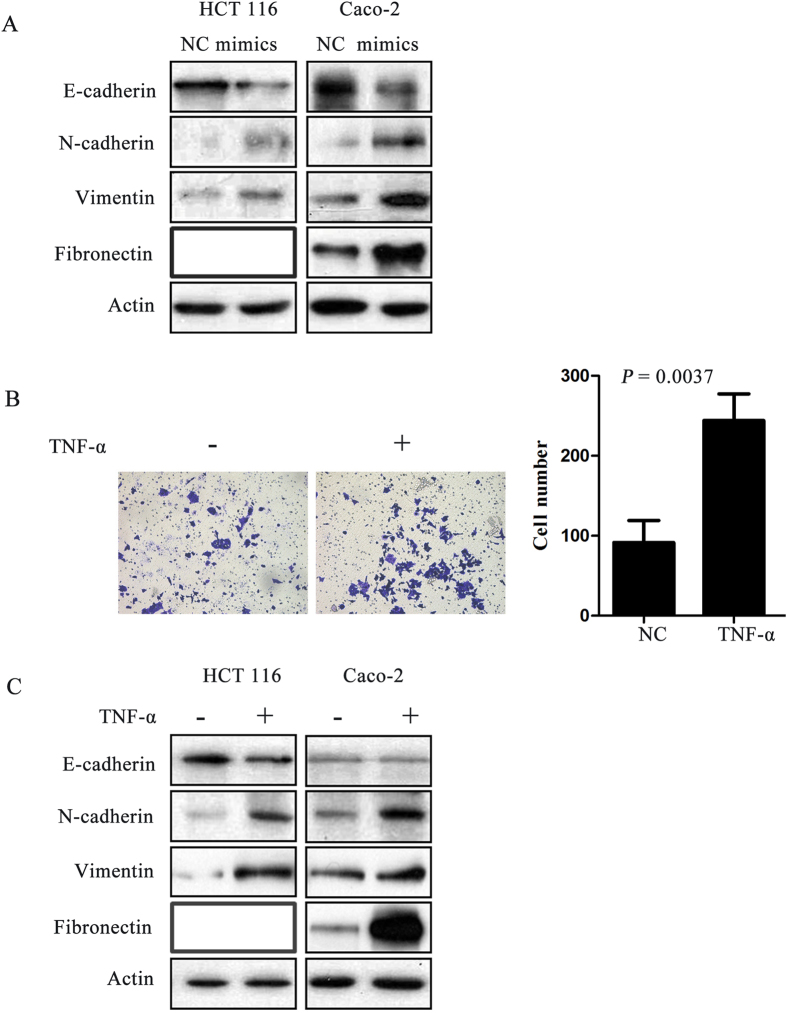
MiR-19a and TNF–α induced EMT in CRC cells. (**A**) Overexpression of miR-19a decreased the expression of the epithelial marker (E-cadherin) but increased the expression of the mesenchymal marker (N-cadherin, vimentin and Fibronectin) in CRC cell lines (The full-length blots were presented in the supplementary Figure S4A). (**B**) TNF-α treatment resulted in a significant increase in cells invasion. (**C**) Western blot analysis showed that this morphological change was associated with the down-regulation of characteristic epithelial protein, E-cadherin, and the up-regulation of characteristic mesenchymal protein N-cadherin, vimentin and Fibronectin (The full-length blots were presented in the supplementary Figure S4C).

EMT has been found to be involved in invasion and metastasis of various cancer cells^8^,^21^,^22^. To better understand whether miR-19a is involved in this progression, epithelial and mesenchymal markers were assessed by western blot analysis of miR-19a mimics and control transfected CRC cells. Overexpression miR-19a in both HCT 116 and Caco-2 cells decreased the levels of the epithelial markers, E-cadherin, whereas increased the mesenchymal markers, vimentin, N-cadherin or Fibronectin. Fibronectin was not detectable in HCT 116 cells (Fig. 4A). The results suggested that miR-19a can induce EMT in CRC cell lines.

### TNF-α induced EMT in CRC cell lines

Previous studies have showed that TNF-α can induce EMT and thereby promote CRC cells invasion and metastasis^21^. The EMT of HCT 116 and Caco-2 cells were observed after stimulation with 10 ng/ml TNF-α for three days. HCT 116 cells resulted in a significant change in morphology, from cobblestone morphology to mesenchymal spindle-like and fusiform features (Supplementary Fig. S3A). The invasion abilities of HCT 116 cells affected by TNF-α were measured by using invasion assays. TNF-α treatment resulted in a significant increase in cells invasion (*P* = 0.0037, Fig. 4B). Western blot analysis showed that this morphological change was associated with the down-regulation of characteristic epithelial protein, E-cadherin, and the up-regulation of characteristic mesenchymal protein N-cadherin, Vimentin or Fibronectin (Fig. 4C). Collectively, these results suggested that TNF-α induces EMT in CRC cell lines.

### MiR-19a was crucial for TNF-α-mediated EMT

**Figure 5:**
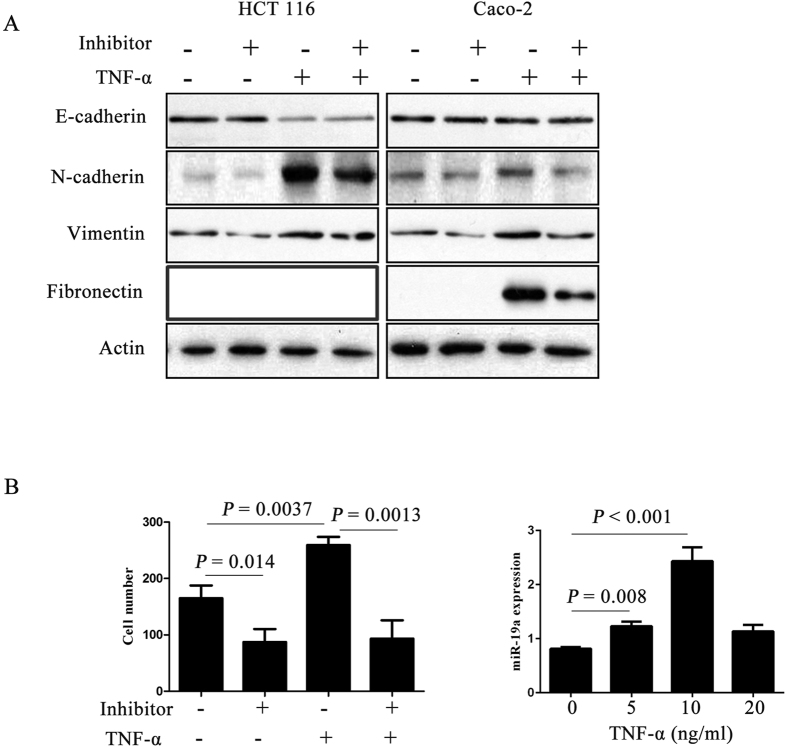
MiR-19a was crucial for TNF-α induced EMT in CRC cells. (**A**) The HCT 116 and Caco-2 cells transfected either miR-19a inhibitor or negative control were treat with or without TNF-α for 48 h. Expression of epithelial and mesenchymal markers was analyzed by western blotting, β-actin servers as the loading control. The HCT 116 and Caco-2 cells transfected with miR-19a inhibitor reversed TNF-α effect on EMT transition (The full-length blots were presented in the supplementary Figure S5). (**B**) The HCT 116 cells transfected either miR-19a inhibitor or negative control with or without TNF-α were subjected to invasion assays. The HCT 116 cells transfected with miR-19a inhibitor reversed TNF-α effect on cell invasion. (**C**) The HCT 116 cells were treated with TNF-α for 72 hours, miR-19a expression was examined by Real-Time PCR.

To better understand the relationship between miR-19a and TNF-α-mediated EMT, HCT 116 cells were transfected with anti-miR-19a oligo or negative control for 24 hours, and then treated with 10 ng/ml TNF-α for 48 hours. Morphological changes were observed under a phase contrast microscope (Supplementary Fig. S3A). The expression of epithelial and mesenchymal markers were detected by western blot analysis and N-cadherin, Vimentin or Fibronectin, was found to decrease in the presence of anti-miR-19a compared to controls in CRC cell lines (Fig. 5A). The effect of miR-19a inhibition on TNF-α-induced invasion was investigated using invasion assays. Compared with negative control, miR-19a inhibition abrogated the abilities of TNF-α to induce spindle-like morphological features (Supplementary Fig. S3). Although E-cadherin levels did not change, miR-19a inhibition dramatically attenuated TNF-α-induced up-regulation of N-cadherin, Fibronectin and Vimentin (Fig. 5A). Consistent with these results, invasion analysis showed that miR-19a inhibition attenuated TNF-α-induced invasion (Fig. 5B). HCT 116 cells were treated with TNF-α for 72 hours, miR-19a expression was examined by Real-Time PCR. TNF-α treatment resulted in a significant increase in miR-19a expression (Fig. 5C). Taken together, these observations demonstrated that miR-19a is essential for TNF-α-mediated EMT in HCT 116 cells.

## Discussion

Metastasis is the main cause of mortality in colorectal cancer. Although many studies showed that miR-19a acts as oncomiR^14^,^18^,^19^,^23^, the function of miR-19a in the metastasis development of CRC remains unclear. This study showed a novel potential molecular mechanism in CRC lymph node metastasis. We found that miR-19a was frequently up-regulated in CRC tissues and a high expression of miR-19a was associated with lymph node metastasis. We demonstrated that miR-19a expression correlated with reduced E-cadherin and increased N-cadherin, Vimentin and Fibronectin expression in CRC cells. In addition, we also found that miR-19a was up-regulated by TNF-α and miR-19a was required for TNF-α-induced EMT in CRC cells. We proposed that miR-19a may serve as a potential marker of lymph node metastasis

Increasing evidences demonstrated that miR-19a acts as an oncomiR in various cancers such as multiple myeloma, breast cancer, and cervical carcinoma^14^,^18^,^23^ and it is up-regulated in lung cancer, gastric cancer and esophageal squamous cell carcinoma (ESCC)^13^,^19^,^22^. In our study, miR-19a was consistently up-regulated in CRC tissues compared with adjacent normal tissues. On the other hand, Zhang *et al.* reported that the expression level of miR-19a had no difference in 13 cases of CRC with distant metastasis^16^. This might be due to the different TNM stage or the limit of sample size. Current evidences suggest several roles of miRNA, including enhancement of colony formation, influence of therapeutic induced cell apoptosis, alternation of drug resistance-related proteins, and promotion of angiogenesis and tumor stem-like cells, however, the mechanisms of miRNA-regulated tumor progression are still largely unknown Here, we showed that overexpression of miR-19a promoted invasion of colon cancer cells, consistent with the findings of Zhang *et al.*^16^, which revealed that overexpressing miR-19a exhibited significantly enhanced invasive ability. Furthermore, we demonstrated that over-expression of miR-19a significant promoted tumorsphere growth, colony formation and drug resistance. This suggested that miR-19a also acts as an oncomiR in CRC^9^,^16^,^24^,^25^.

In spite of the growing evidences high-lighting the important role of miR-19a in cancer, none of previous study systematically investigated the role of the miR-19a in the development of lymph node metastasis in human CRC. Our present study next focused on the potential relationship between the expression of miR-19a and various clinicopathological characteristics, particularly lymph node metastasis. The results showed that high levels of miR-19a were significantly correlated with TNM stage, which is similar to the findings in bladder cancer and gastric cancer^26^,^27^. Interestingly, our results showed that miR-19a was up-regulated in CRC patients presenting lymph node metastasis. Together, miR-19a was up-regulated in CRC tissues compared with adjacent normal tissues and its overexpression was associated with lymph node metastasis, suggesting that its up-regulation was acquired in the course of tumor progression and in particular, during the acquisition of metastatic potential. We also performed the association study to evaluate the correlation of miR-19a expression with the metastatic potential using the TCGA data portal. In this patient subpopulation, miR-19a was consistently up-regulated in rectal cancer patients presenting lymph node metastasis, supporting the results obtained in our study. Taken together, these results indicate that miR-19a could serve as a potential biomarker of lymph node metastasis in CRC.

One of the key molecular steps in the process of metastasis includes EMT, which allows epithelial cells to acquire mesenchymal-like properties, invade the surrounding tissue, migrate into lymphatic or vascular tissue and metastasize to distant sites. Recent studies suggested that miRNAs have emerged as crucial mediators in regulating EMT-related genes. Hur *et al.* found that miR-200c plays an important role in mediating EMT and metastatic behavior in the colon cancer^28^. EMT-related genes (ZEB1, ETS1 and FLT1) are clarified to be the target genes of miR-200c in CRC cell lines. Increased expression of miR-200c results in the negative regulation of its gene targets, which in turn down-regulates E-cadherin and elevates vimentin expression to trigger an EMT switch in CRC cells. Kumarswamy *et al.* reported that in non-small cell lung cancer, miR-30a inhibits invasion and metastasis through targeting Snai1, a known transcriptional repressor of E-cadherin and modulator of EMT^29^. In our study, we discovered that the levels of the epithelial markers, E-cadherin, decreased and 3 mesenchymal markers (N-cadherin, Vimentin, and Fibronectin) increased in CRC cells after over-expression of miR-19a. The results suggested that miR-19a induced EMT in colon cancer cells. This finding is consistent with clinical observations, which revealed that miR-19a was higher expression in cancer with lymph node metastasis than those without lymph node metastasis. EMT has also been shown to promote stem-like cancer cells that may endow tumor initiating cells with traits necessary for metastasis formation^30^,^31^, which provides a possible explanation for miR-19a function of tumorsphere growth, colony formation and drug resistance.

Several inflammatory mediators such as TGF-β, hypoxia and IL-6 can trigger EMT^14^,^19^. Accumulating evidences indicated that inflammatory mediators trigger EMT and invasion through interacting with miRNAs. For instance, miR-30a is consistently down-regulated after TGF-β treatment and miR-30a can inhibit TGF-β-induced loss of E-cadherin^29^,^32^. Bai *et al.* found that miR-200c suppressed invasion of breast cancer cells by targeting ZNF217, a transcriptional activator of TGF-β, and ZEB1, and a known mediator of TGF-β signaling^33^. Previous studies have showed that TNF-α expression is also associated with tumor progression of colorectal adenocarcinomas and high TNF-α expression is strongly associated with tumor recurrence in CRC patients with positive lymph node metastases^20^. In this study, we found that pro-inflammatory cytokine TNF-α can induce EMT in CRC cells and promote CRC cells invasion, which was consistent with the findings from Wang *et al.* that TNF-α induces EMT in human HCT 116 cells and thereby promotes CRC invasion and metastasis^21^. Further results showed that miR-19a inhibition attenuated TNF-α-induced up-regulation of mesenchymal protein and invasion. Subsequent analysis indicated that miR-19a was consistently up-regulated after TNF-α treatment. This suggested that miR-19a was essential for TNF-α-mediated EMT and provided mechanistic insight into the role of miR-19a in mediating some of the TNF-α mediated EMT activity. Considering, TNF-α has been reported previously to be a miR-19a gene target in ESCC^34^ and ulcerative colitis^35^, we had conducted the western blot assay to determine whether TNF-α would be a target in CRC cells. Ectopic miR-19a expression decreased the expression of TNF-α in both HCT 116 and Caco-2 cells (Supplementary Fig. S4). Feedback loops feature in a number of genetic pathways involving miRNAs, where they seem to enhance the robustness of gene regulation^36^. In our study, miR-19a was consistently up-regulated after TNF-α treatment, whereas TNF-α is negatively regulated by miR-19a. Altogether, the relationship between TNF-α and miR-19a should be further elucidated.

In conclusion, our findings demonstrated a potential role of miR-19a in CRC lymph node metastasis. We found that miR-19a was frequently up-regulated in CRC tissues, high expression of miR-19a was associated with lymph node metastasis, and miR-19a overexpression promoted invasion of human CRC cell lines. We also showed that miR-19a was essential for TNF-α-mediated EMT in CRC cells. Given the widely accepted links between miRNAs and the pathogenesis of CRC, these discoveries provided a better understanding for CRC lymph node metastasis.

## Materials and Methods

### Tissue specimens and patient information

The procedure of human sample collection was approved by the Ethical Committee of Sun Yat-sen University (Guangzhou, China), and all patients signed informed consent for the collection and use of their tissues for this study. All experimental protocols were carried out in accordance with the approved guidelines and were approved by the Ethical Committee of Sun Yat-sen University (Guangzhou, China). In this study, 275 formalin-fixed, paraffin-embedded samples of CRC were obtained from the tumor bank of the Department of Pathology of the First Affiliated Hospital, Sun Yat-sen University (Guangzhou, China). These 275 patients, who had been diagnosed with CRC and underwent initial surgical resection for CRC between January 2000 and November 2006, were contacted for a follow-up by telephone or letters from surgery until April 2010 to collect general information, pathology reports, and information regarding the patients’ condition after surgery. Frozen fresh CRC tissues and paired non-tumor tissues were obtained from the surgical pathology archives of the gastrointestinal surgery department at Sun Yat-sen Memorial Hospital.

### Human cell lines

HCT 116 and Caco-2 were purchased from Culture Collection of Chinese Academy of Science (Shanghai, China). HCT 116 and Caco-2 were cultured in RPMI 1640/DMEM supplemented with 10% FBS and 1% penicillin-streptomycin at 37 °C in 5% CO_2_.

### Lentivirus packaging and transduction

MiR-19a precursor sequences was amplified from human genomic DNA and cloned into the AgeI and EcoRI site of the lentiviral vector GV254 (Genchem) (pGV-miR-19a). Virus packaging was performed in 293T cells. pGV254-miR-19a, pHelper 1.0 and pHelper 2.0 cotransfections were using Lipofectamine 2000 (Invitrogen) according to the manufacturer’s instruction. 293T cells were cultured in DMEM in a 37 °C incubator with 5% CO_2_. Eight hours after transfection, the medium was refreshed. 48 hours later, the supernatant was harvested. Empty lentiviral vector GV254 was used as the negative control (pGV-NC). HCT 116 and Caco-2 cells were infected with either pGV-miR-19a or pGV-miR-NC in the presence of 5 μg/ml polybrene for 12 hours, and the medium was refreshed. 72 hours post the infection, the efficiency of infection was measured under a fluorescent microscope.

### Analysis of miRNA expression using TaqMan RT-PCR

Total RNA from cultured cells and tissues was extracted using Trizol (Invitrogen). Expression of mature miR-19a was analyzed using the TaqMan miRNA Assay (Applied Biosystems Inc., Foster City, CA). Expression of RNU6B (Applied Biosystems Inc., Foster City, CA) was used as an endogenous control. All the experiments were done in triplicate. Real-Time PCR was performed using an Applied Biosystems 7500 Real-Time PCR system (Applied Biosystems). MiR-19a expression △Ct values from each sample were calculated by normalizing with an internal control (RNU6B), and relative expressions were calculated using the formula 2^−△△Ct^ values. Differences between miRNA expression levels among two groups were evaluated using a t-test; *P*-values < 0.05 were considered significant. Statistics were performed with SPSS.

### *In situ* hybridization

*In situ* hybridization (ISH) was performed using a miRNA-19a probe from Exiqon (miRCURY LNA™ Detection probe, 250 pmol, 5′-DIG labeled). Detection of the probe was carried out using anti-digoxgenin-AP (Roche), and the hybridized probes were detected by applying BCIP/NBT Alkaline Phosphatase Color Development Kit. No probe controls were included for each hybridization procedure. Images were taken by the Leica DMI 4000B inverted microscope (Leica Micro-systems, Wetzlar, Germany). *In situ* hybridisation staining of the image was analyzed by using the Image Pro-Plus (version 5.0, Media Cybernetics, Silver Spring, USA) introduced by Xavier^37^. In brief, the tumor area was selected as the area of interest (AOI), and the area sum and integrated optical density (IOD) of the AOI were selected as the measurement parameters. MiR-19a expression index was equaled the quotient between the IOD and the total area of AOI. Finally, the mean expression index for each duplicate was used for statistical analysis.

### TCGA dataset

The TCGA IlluminaHiseq miRNASeq data set with clinical information was downloaded on 06/19/2015. The set included 218 colon adenocarcinoma and 93 rectum adenocarcinoma microRNA data sets.

### Invasion assay

Transwell chambers precoated with Matrigel (BD Bioscience, San Jose, CA, USA) were used to perform the invasion assay. Cells were cultured in serum-free medium with or without 10 ng/ml TNF-α (PeproTech) in the upper chambers of a Transwell (BD) plate (5 × 10^4^ cells per chamber), which are separated from the lower chambers with permeable 8.0 mm polycarbonate membranes; medium containing 10% FBS served as the attractant in the lower chambers. After 48 hours, the cells were fixed with 4% Polyoxymethylene and stained with crystal violet. Non-migrating cells on the upper side of the membrane were gently wiped off, and the stained cells on the lower side were observed under a microscope. The number of migrating cells in five fields per chamber was counted and average values were calculated.

### Transient transfection of miRNA mimics

MiR-19a mimics, inhibitor or negative control were transfected in the different CRC cell lines using Lipofectamine 2000 (Invitrogen), according to the manufacturer’s recommendations. RNA and protein analyses were performed within 72 hours from transfection. In each experiment, the extent of miR-19a expression was assessed by Real-Time PCR.

### Soft agar colony formation assay

5 × 10^3^ cells were mixed with 0.3% low melting agar in DMEM supplemented with 10% FBS and plated on 0.5% agar-coated 6-well plate. Plates were incubated at 37 °C in humidified incubator for 15 days. Media was changed on the cells 1–2 times per week with fresh cell culture media. Plates were stained with 0.2 ml of 0.005% Crystal Violet for more than 1 hour. The number of colonies was counted under phase contrast microscopy with low magnification.

### Tumorsphere formation assay

Cells were suspended in serum-free culture medium DMEM containing 20 ng/ml EGF, 20 ng/ml bFGF, and 1 × B27, and then plated in 6-well ultra-low attachment plates at a concentration of 5 × 10^3^ cells per well. 15 days later, plates were analyzed for tumorshpere formation and were quantified using inverted microscope at 100×, 200× magnifications.

### Western blot analysis

The procedures for Western blotting were performed as described previously. Briefly, equal amounts of protein were separated by 10% sodium dodecyl sulphate-polyacrylamide gel electrophoresis (SDS-PAGE) and transferred to a polyinylidene fluoride (PVDF) membrane (Millipore). The membranes were then blocked with 5% skim-milk for 1 hour at room temperature and incubated with primary antibodies (E-cadherin, N-cadherin, Vimentin, Fibronection and ß-actin) overnight at 4 °C. The next day, after incubating with horseradish peroxidase-conjugated secondary antibodies for 1 hour at room temperature, the signals were detected using a chemiluminescence detection kit ECL (Santa Cruz biotechnology).

### Statistical analysis

Statistical analysis was done using the SPSS software (version 17.0, SPSS Inc, Chicago, USA). Statistical significance was determined using Student’s t-test or Mann-Whitney U test as appropriate. The correlations between miR-19a expression levels and the potential factors in CRC patients were evaluated by performing Chi-square Test. Survival curves were generated using the Kaplan-Meier method and assessed using the log-rank test. The independent prognostic factors were identified by performing Multivariate Cox regression analysis. The optimal single cut-point for the survival curve was determined using X-tile software (version 3.6.1, Yale University School of Medicine, New Haven, USA). All *P* - values were two sided and *P* < 0.05 was considered statistically significant.

## Additional Information

**How to cite this article**: Huang, L. *et al.* Hsa-miR-19a is associated with lymph metastasis and mediates the TNF-a induced epithelial-to-mesenchymal transition in colorectal cancer. *Sci. Rep.* **5**, 13350; doi: 10.1038/srep13350 (2015).

## Supplementary Material

Supplementary Information

## Figures and Tables

**Table 1:** Correlation between miR-19a expression and clinical parameters in 275 CRC patients.

**Factors**	**n**	**Low miR-19a expression (%)**	**High miR-19a expression (%)**	***P*value**
Age				0.873
<60	142	109(76.8)	33(23.2)	
≥60	133	101(75.9)	32(24.1)	
Gender				0.759
Male	152	115(75.7)	37(24.3)	
Female	123	95(77.2)	28(22.8)	
Tumor size				0.239
<5 cm	139	102(73.4)	37(26.6)	
≥5 cm	136	108(79.4)	28(20.6)	
Tumor location				0.812
Colon	132	102(77.3)	30(22.7)	
Rectum	142	108(76.1)	34(23.9)	
Relapse				0.112
Yes	38	33(86.8)	5(13.2)	
No	233	175(75.1)	58(24.9)	
TNM stage				0.008*
I	49	39(79.6)	10(20.4)	
II	99	81(81.8)	18(18.2)	
III	102	67(65.7)	35(34.3)	
IV	25	23(92.0)	2(8.0)	
pT				0.805
T1 + T2	58	45(77.6)	13(22.4)	
T3 + T4	217	165(76.0)	52(23.9)	
pN				0.018*
N0	156	128(82.1)	28(17.9)	
N1 + N2	112	78(69.6)	34(30.4)	
pM				0.054
M0	250	187(74.8)	63(25.2)	
M1	25	23(92.0)	2(8.0)	

*P < 0.05, Chi-square test.

**Table 2:** Univariate and multivariate analyses of various potential prognostic factors in 275 CRC patients.

**Factors**	**Univariate analysis**		**Multivariate analysis**	
	**HR**^a^ **(95%CI**^b^)	***P***	**HR**^a^ **(95%CI**^b^)	***P***
Age	1.00(0.64, 1.54)	0.983		
Gender	1.06(0.68, 1.65)	0.792		
Tumor size	1.15(0.74, 1.79)	0.523		
Tumor location	0.61(0.39, 0.95)	0.030	0.63(0.40, 0.98)	0.042*
Relapse	3.89(2.41, 6.27)	<0.001	2.56(1.54, 4.27)	<0.001*
TNM stage	3.03(1.91, 4.81)	<0.001	2.41(1.47, 3.96)	<0.001*
miR-19a expression	0.68(0.38, 1.19)	0.171		

^a^HR, hazard ratio.

^b^CI, confidence interval.

*P < 0.05.
