# Supplementary material for: Hsa-miR-19a is associated with lymph metastasis and mediates the TNF-α induced epithelial-to-mesenchymal transition in colorectal cancer
Source: Sci Rep. 2015 Aug 25;5:13350. doi: 10.1038/srep13350 (PMC5388049; doi:10.1038/srep13350)
Supplement: Supplementary Information [file srep13350-s1.pdf]

Hsa-miR-19a is associated with lymph metastasis and mediates the TNF-α induced epithelial-to-mesenchymal transition in colorectal cancer

Lanlan Huang[1, 2, 3, 4], Xiaoyan Wang[1, 2, 3, 4], Chuangyu Wen[1, 2, 3, 4], Xiangling Yang[1, 2, 3, 4], Minmin Song[1, 2, 3, 4], Junxiong Chen[1, 2, 3, 4], Chenliang Wang[1, 2, 3, 4], Bo Zhang[1, 2], Lei Wang[1, 2], Aikichi Iwamoto[5], Jianping Wang[1, 2*], Huanliang Liu[1, 2, 3, 4*]

[1]Guangdong Institute of Gastroenterology and the Sixth Affiliated Hospital, [2]Guangdong Key Laboratory of Colorectal and Pelvic Floor Diseases, [3]Institute of Human Virology, [4]Key Laboratory of Tropical Disease Control (Ministry of Education), Sun Yat-sen University, Guangzhou, China, [5]Advanced Clinical Research Center, Institute of Medical Science, University of Tokyo, Tokyo, Japan

Corresponding author:

Huanliang Liu. 26 Yuancun Er heng Road, Guangzhou, Guangdong 510655, China. TEL: +86-20-38455492; E-mail address: liuhuanl@mail.sysu.edu.cn; huanliang.liu@gmail.com

Jianping Wang. 26 Yuancun Er heng Road, Guangzhou, Guangdong 510655, China. TEL: +86-20-38254419; E-mail address: wangjpgz@yahoo.com.cn

Supplementary Materials

Table S1. Clinical and histopathological information of the 11 cases of CRC.

Case no.	Gender (M/F)	Age (years)	Location	Lymph node invasion	distant metastasis	TNM stage
1	F	70	Sigmoid colon	Yes	No	III
2	F	37	Transverse colon	No	No	II
3	F	73	Rectum	No	No	II
4	F	75	Rectum	Yes	No	III
5	M	65	Sigmoid colon	Yes	No	III
6	F	79	Right colon	No	No	II
7	M	59	Sigmoid colon	Yes	No	III
8	M	52	Rectum	Yes	No	III
9	M	85	Right colon	No	No	II
10	M	51	Left colon	Yes	No	III
11	F	85	Sigmoid colon	Yes	No	III

M, male; F, female.

Table S2. Patient demographic and clinical characteristics of 275 CRC patients.

**Characteristics**	**of cases**
**Age (years)**	
<60	142 (51.64%)
≥60	133 (48.36%)
**Gender**	
Male	152 (55.27%)
Female	123 (44.73%)
**Tumor size**	
<5cm	139 (50.55%)
≥5cm	136 (49.45%)
**Tumor location**	
Colon	132 (48%)
Rectum	142 (51.64%)
Missing data	1 (0.36%)
**Differentiation**	
Well	21 (7.64%)
Moderate	219 (79.64%)
Poor	31 (11.27%)
Undifferentiation	1 (0.36%)
Missing data	2 (0.73%)
**Primary tumor (pT)**	
T1	6 (2.18%)
T2	52 (18.91%)
T3	198 (72%)
T4	19 (6.91%)
**Regional lymph node (pN)**	
N0	156 (56.73%)
N1	77 (28%)
N2	35 (12.8%)
Missing data	7 (2.5%)
**Distant Metastasis (pM)**	
M0	250 (90.91%)
M1	25 (9.09%)
**TNM stage**	
I	49 (17.82%)
II	99 (36%)
III	102 (37.09%)
IV	25 (9.09%)
**Relapse**	
Yes	38 (13.82%)
No	233 (84.73%)
Missing data	4 (1.45%)

Table S3. Patient demographic and clinical characteristics of 218 colon cancer patients.

**Characteristics**	**of cases**
**Age (years)**	
<60	65 (29.8%)
≥60	153 (70.2%)
**Gender**	
Male	124 (56.9%)
Female	94 (43.1%)
**TNM stage**	
Ⅰ	31 (14.2%)
Ⅱ	89 (40.8%)
Ⅲ	64 (29.4%)
Ⅳ	26 (11.9%)
Missing data	8 (3.7%)
**Primary tumor (pT)**	
TX	1 (0.5%)
T1	5 (2.3%)
T2	31 (14.2%)
T3	156 (71.6%)
T4	25 (11.5%)
**Regional lymph node (pN)**	
NX	1 (0.5%)
N0	128 (58.7%)
N1	53 (24.3%)
N2	36 (16.5%)
**Distant Metastasis (pM)**	
MX	36 (16.5%)
M0	152 (69.7%)
M1	26 (11.9%)
Missing data	4 (1.8%)

Table S4. Patient demographic and clinical characteristics of 93 rectal cancer patients.

**Characteristics**	**of cases**
**Age (years)**	
<60	37 (39.8%)
≥60	56 (60.2%)
**Gender**	
Male	50 (53.8%)
Female	43 (46.2%)
Missing data	78 (83.9%)
**TNM stage**	
Ⅰ	13 (14.0%)
Ⅱ	25 (26.9%)
Ⅲ	34 (36.6%)
Ⅳ	16 (17.2%)
Missing data	5 (5.4%)
**Primary tumor (pT)**	
T1	5 (5.4%)
T2	14 (15.1%)
T3	63 (67.7%)
T4	10 (10.7%)
Missing data	1 (1.1%)
**Regional lymph node (pN)**	
NX	2 (2.2%)
N0	40 (43.0%)
N1	29 (31.2%)
N2	21 (22.6%)
Missing data	1 (1.1%)
**Distant Metastasis (pM)**	
MX	14 (15.1%)
M0	64 (68.8%)
M1	13 (14.0%)
Missing data	2 (2.2%)

Table S5. Correlation between miR-19a expression and clinical parameters in 218 colon cancer patients.

Factors	n	Low miR-19a expression (%)	High miR-19a expression (%)	*P* - value
**Age**				0.104
<60	65	47 (72.3)	18 (27.7)	
≥60	153	93 (60.8)	60 (39.2)	
**Gender**				0.108
Male	124	74 (59.7)	50 (40.3)	
Female	94	66 (70.2)	28 (29.8)	
**TNM stage**				0.299
Ⅰ	31	21 (67.7)	10 (32.3)	
Ⅱ	89	54 (60.7)	35 (39.3)	
Ⅲ	64	42 (65.6)	22 (34.4)	
Ⅳ	26	21 (80.8)	5 (19.2)	
**pT**				0.433
T1+T2	36	21 (58.3)	15 (41.7)	
T3+T4	181	118 (65.3)	63 (34.8)	
**pN**				0.302
N0	128	79 (61.7)	49 (38.3)	
N1+N2	89	61 (68.5)	28 (31.5)	
**pM**				0.062
M0	152	94 (61.8)	58 (38.2)	
M1	26	21 (80.8)	5 (19.2)	

Table S6. Correlation between miR-19a expression and clinical parameters in 93 rectal cancer patients.

**Factors**	**n**	**Low miR-19a expression (%)**	**High miR-19a expression (%)**	***P* - value**
**Age**				0.638
<60	37	16 (43.2)	21 (56.8)	
≥60	56	27 (48.2)	29 (51.8)	
**Gender**				0.086
Male	50	19 (38.0)	31 (62.0)	
Female	43	24 (55.8)	19 (44.2)	
**TNM stage**				0.104
Ⅰ	13	7 (53.8)	6 (46.2)	
Ⅱ	25	15 (60.0)	10 (40.0)	
Ⅲ	34	10 (29.4)	24 (70.6)	
Ⅳ	16	8 (50.0)	8 (50.0)	
**pT**				0.728
T1+T2	19	8 (42.1)	11 (57.9)	
T3+T4	73	34 (46.6)	39 (53.4)	
**pN**				0.004
N0	40	25 (62.5)	15 (37.5)	
N1+N2	50	16 (32.0)	34 (68.0)	
**pM**				0.579
M0	64	30 (46.9)	34 (53.1)	
M1	13	5 (38.5)	8 (61.5)	

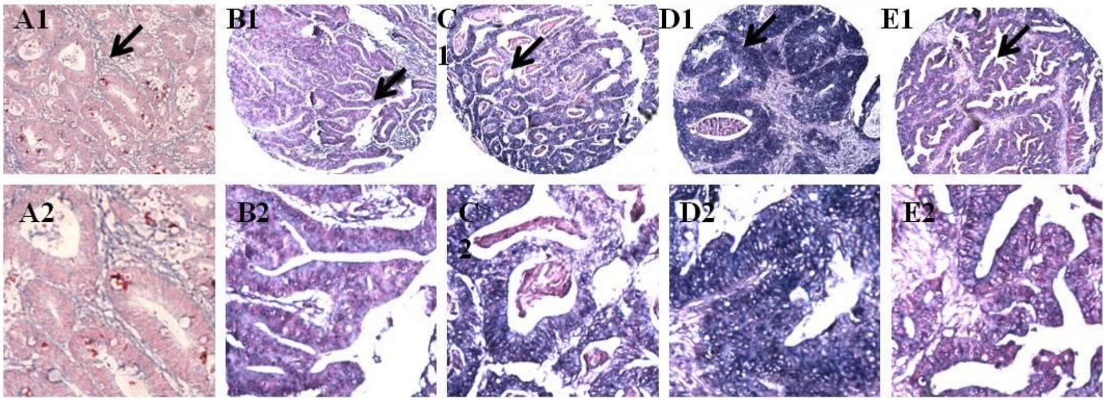

Fig. S1 Expression of miR-19a in CRC using *in situ* hybridisation analysis.

Tissue sections were incubated with DIG–labelled LNA probe to miR-19a. The positive staining of tissue was expressed as blue–violet. No in situ hybridization signal was obtained in the absence of DIG–labeled probe (A1, A2). Weak staining of miR-19a was detected in stage Ⅰ (B1, B2) and Ⅳ (E1, E2), stage Ⅱ moderately positive expression of miR-19a (C1, C2), stage Ⅲ strongly positive expression of miR-19a (D1, D2).

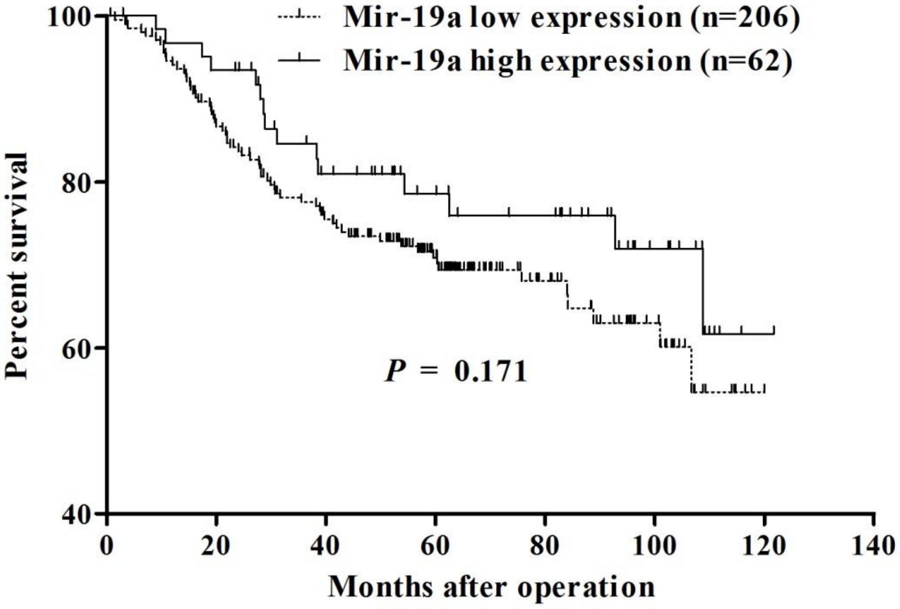

Fig. S2 Association between miR-19a expression in colorectal cancer and overall survival in 268 CRC patients.

In situ hybridization from paraffin blocks of CRC specimens followed by analysis of miR-19a levels. The Kaplan-Meier curve and log-rank test showed that miR-19a expression wasn’t associated with survival (*P* = 0.171).

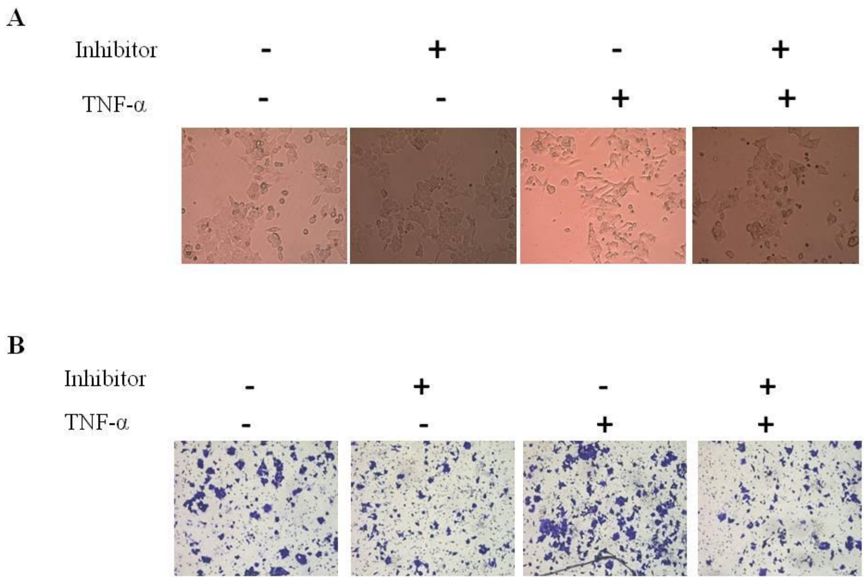

Fig. S3 MiR-19 inhibitor reversed the TNF-α induced EMT transition.

(A) The HCT 116 cells transfected either miR-19a inhibitor or negative control were treated with or without TNF-α. Cell morphology was examined and photographed.

(B) The HCT 116 cells transfected either miR-19a inhibitor or negative control with or without TNF-α were subjected to invasion assays. The HCT 116 cells transfected with miR-19a inhibitor reversed TNF-α effect on cell invasion.

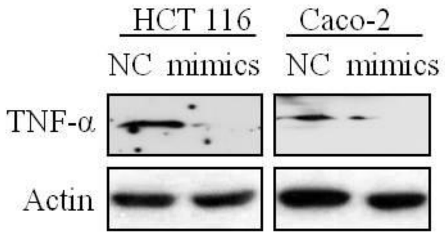

Fig. S4 miR-19a down-regulated the expression of TNF-α.

HCT 116 and Caco-2 cells were transfected with miR-19a mimics or negative control. Western blot analysis showed that miR-19a down-regulated the expression of TNF-α.

Supplementary information

Full-length blots:

Figure S4 A

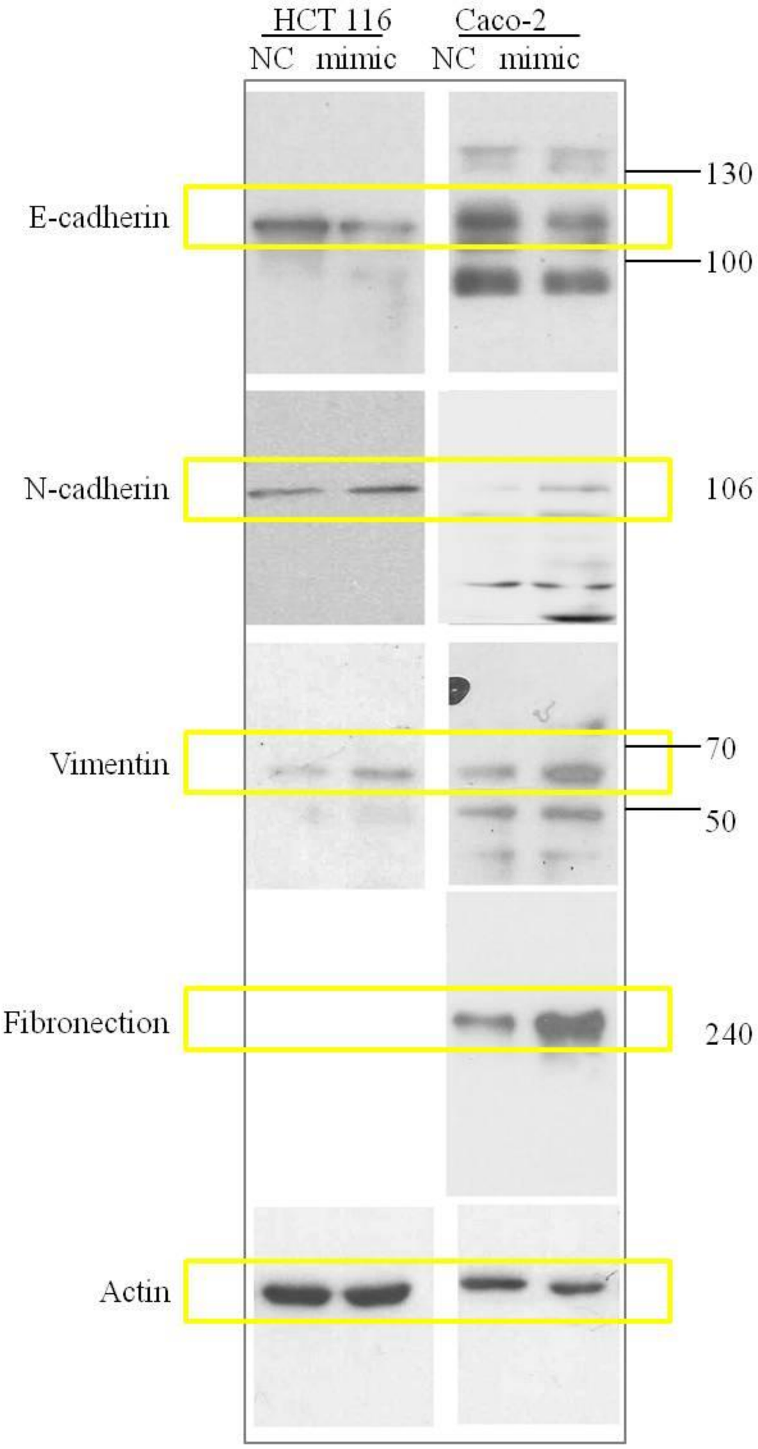

Figure S4 C

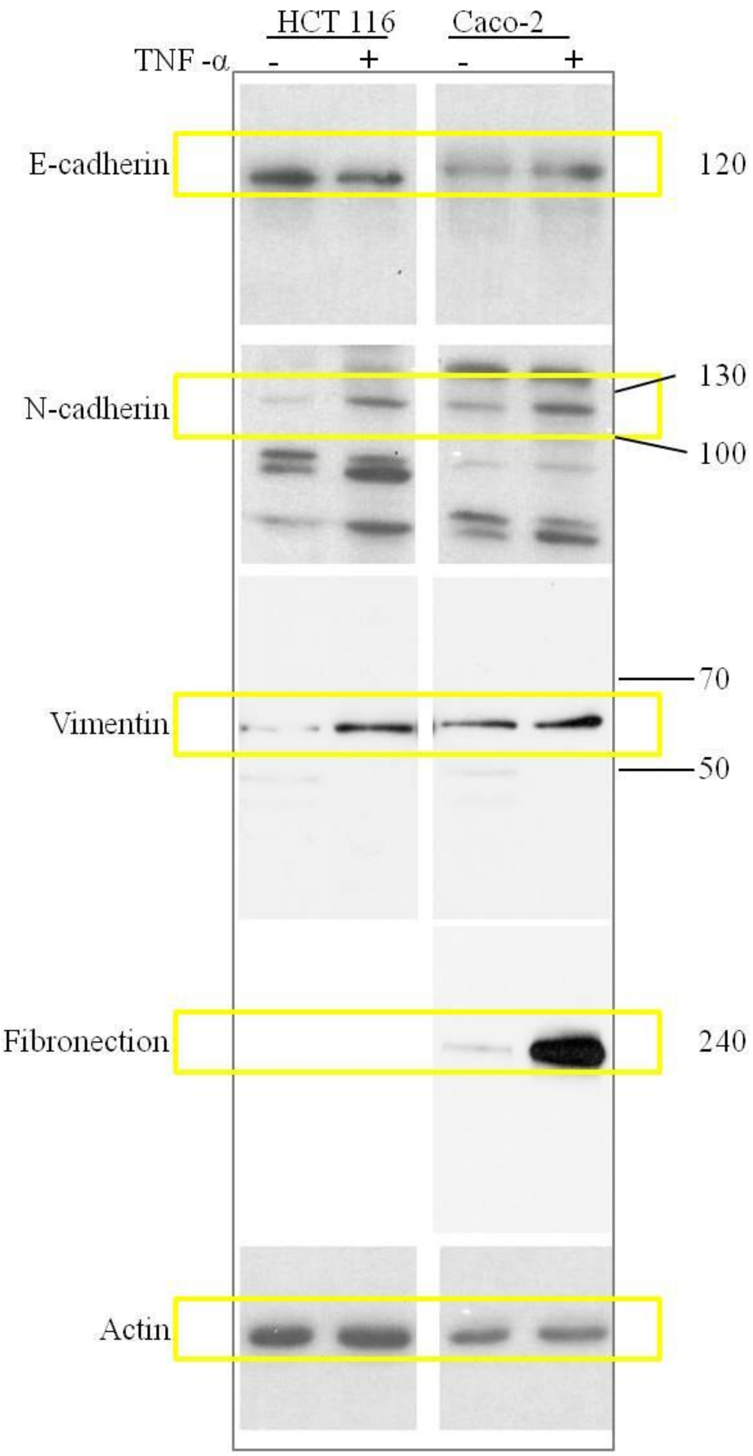

HCT 116
Caco-2
TNF -α
-
+
-
+
E-cadherin
120
N-cadherin
130
100
Vimentin
70
50
Fibronection
240
Actin

Figure S5

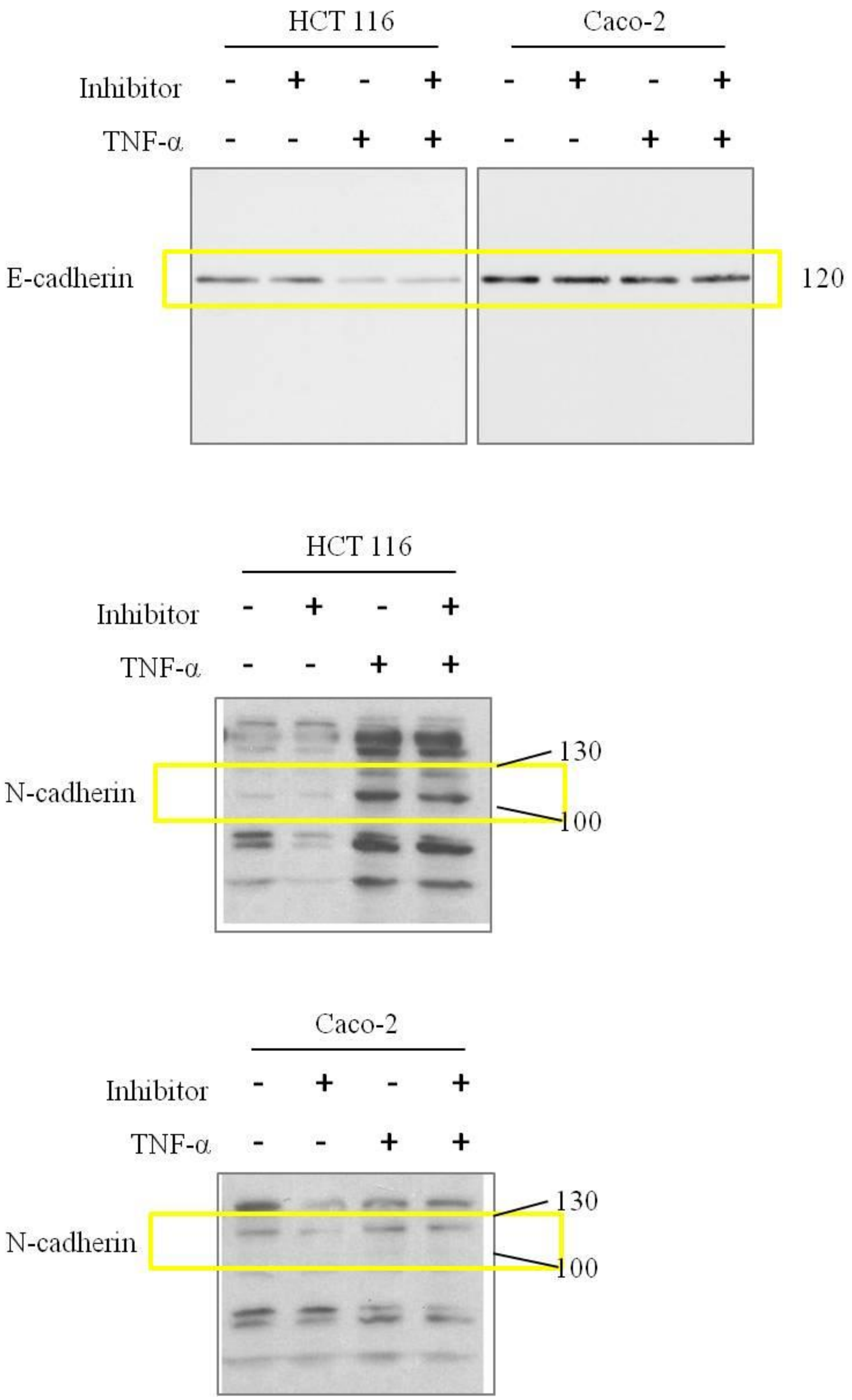
HCT 116
Caco-2
Inhibitor - + - + - + - +
TNF-α - - + + - - + +
E-cadherin
120
HCT 116
Inhibitor - + - +
TNF-α - - + +
N-cadherin
130
100
Caco-2
Inhibitor - + - +
TNF-α - - + +
N-cadherin
130
100

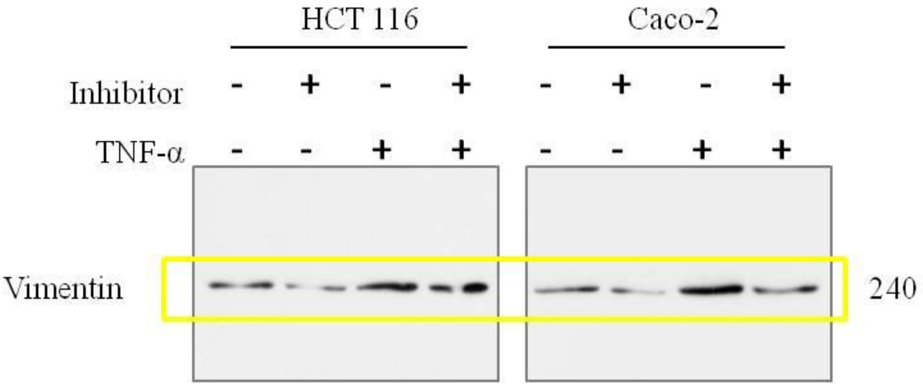
HCT 116
Caco-2
Inhibitor - + - + - + - +
TNF-α - - + + - - + +
Vimentin
240

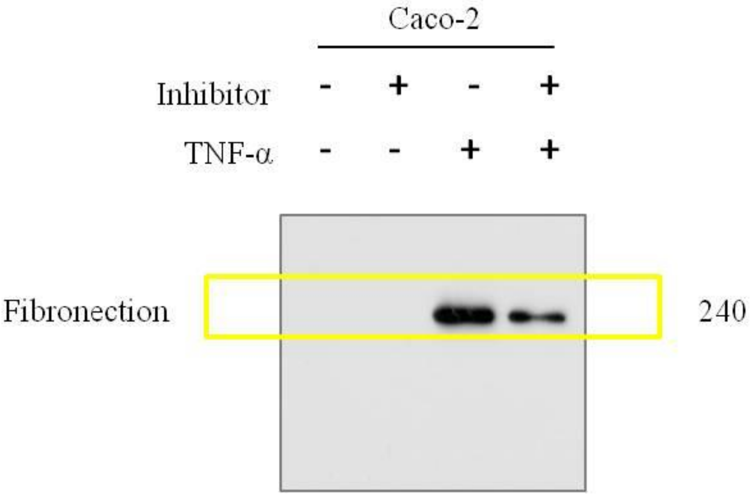
Caco-2
Inhibitor - + - +
TNF-α - - + +
Fibronection
240

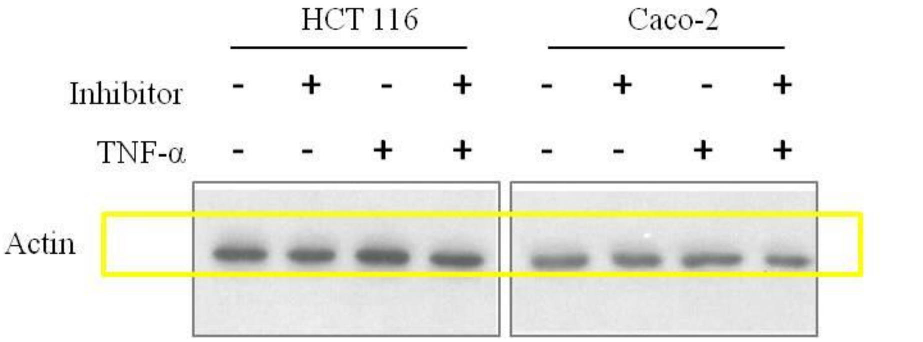
HCT 116
Caco-2
Inhibitor - + - + - + - +
TNF-α - - + + - - + +
Actin
